# Identification of Immune-Associated Genes in Diagnosing Aortic Valve Calcification With Metabolic Syndrome by Integrated Bioinformatics Analysis and Machine Learning

**DOI:** 10.3389/fimmu.2022.937886

**Published:** 2022-07-04

**Authors:** Yufei Zhou, Wenxiang Shi, Di Zhao, Shengjue Xiao, Kai Wang, Jing Wang

**Affiliations:** ^1^ Department of Cardiology, Shanghai Institute of Cardiovascular Diseases, Zhongshan Hospital and Institutes of Biomedical Sciences, Fudan University, Shanghai, China; ^2^ Department of Pediatric Cardiology, Xinhua Hospital, The Affiliated to Shanghai Jiao Tong University School of Medicine, Shanghai, China; ^3^ Department of Cardiology, Zhongda Hospital, School of Medicine, Southeast University, Nanjing, China; ^4^ Department of Cardiology, The First Affiliated Hospital, Zhejiang University School of Medicine, Hangzhou, China; ^5^ Department of Geriatric Medicine, The Affiliated Jiangning Hospital With Nanjing Medical University, Nanjing, China

**Keywords:** aortic valve calcification, metabolic syndrome, differentially expressed genes, machine learning, immune infiltration, diagnosis

## Abstract

**Background:**

Immune system dysregulation plays a critical role in aortic valve calcification (AVC) and metabolic syndrome (MS) pathogenesis. The study aimed to identify pivotal diagnostic candidate genes for AVC patients with MS.

**Methods:**

We obtained three AVC and one MS dataset from the gene expression omnibus (GEO) database. Identification of differentially expressed genes (DEGs) and module gene *via* Limma and weighted gene co-expression network analysis (WGCNA), functional enrichment analysis, protein–protein interaction (PPI) network construction, and machine learning algorithms (least absolute shrinkage and selection operator (LASSO) regression and random forest) were used to identify candidate immune-associated hub genes for diagnosing AVC with MS. To assess the diagnostic value, the nomogram and receiver operating characteristic (ROC) curve were developed. Finally, immune cell infiltration was created to investigate immune cell dysregulation in AVC.

**Results:**

The merged AVC dataset included 587 DEGs, and 1,438 module genes were screened out in MS. MS DEGs were primarily enriched in immune regulation. The intersection of DEGs for AVC and module genes for MS was 50, which were mainly enriched in the immune system as well. Following the development of the PPI network, 26 node genes were filtered, and five candidate hub genes were chosen for nomogram building and diagnostic value evaluation after machine learning. The nomogram and all five candidate hub genes had high diagnostic values (area under the curve from 0.732 to 0.982). Various dysregulated immune cells were observed as well.

**Conclusion:**

Five immune-associated candidate hub genes (*BEX2*, *SPRY2*, *CXCL16*, *ITGAL*, and *MORF4L2*) were identified, and the nomogram was constructed for AVC with MS diagnosis. Our study could provide potential peripheral blood diagnostic candidate genes for AVC in MS patients.

## 1. Introduction

Aortic valve (AV) calcification (AVC) is the most common valvular cardiac disease in the aging population of the developed world. It has a high global prevalence; approximately 12.6 million cases were reported in 2017 with an estimated 102,700 deaths (1, 2). AVC is induced by several risk factors, including genetic mutations, hyperlipidemia, hyperglycemia, and infection (3). Metabolic syndrome (MS) is a pathologic condition comprised of hypertension, hyperlipidemia, abdominal obesity, and insulin resistance (4). Therefore, patients with MS have an amplified risk of AVC but also progresses rapidly from mild to severe (5). Both AVC and MS are progressive diseases constituting a global health burden for the aging population.

Previous studies have proved that early endothelial inflammation and dysfunction take part in the initiation of AVC (6). Several risk factors, including smoking, diabetes, and hyperlipidemia, contribute to AVC, at least in part, through pro-inflammatory molecules and lipid deposition (7). Furthermore, inflammation reaction also exists in late-stage AVC (8). According to multiple pieces of evidence, immune cells play a crucial role in the physiological dysfunction associated with MS, as well as the pathophysiology and development of subsequent chronic diseases (9, 10). As a result, immune filtration and related pro-inflammatory molecules may be useful in the early diagnosis of AVC patients with MS.

It is well recognized that MS can accelerate the AVC proceeding. After the onset of valve disease symptoms, the prognosis is dismal. Patients usually do not seek medical evaluation for AVC until they exhibit symptoms. Therefore, it is crucial to discover sensitive and specific diagnostic tools for early-stage AVC prior to irreversible heart injury, particularly for MS patients who may be insensitive to aorta stenosis (AS) symptoms. Proteomics and sequencing tools provide an opportunity for identifying potential novel biomarkers and their roles in diverse diseases (11). Machine learning is gradually maturing in bioinformatics applications and can be used to excavate underlying mechanisms, prospective biomarkers, and therapeutic targets for a variety of diseases (12).

To the best of our knowledge, limited research has been conducted on the identification of immune-associated diagnostic candidates for AVC with MS, as well as the machine learning application for AVC diagnosis. Here, we first downloaded three AVC and one MS datasets from the gene expression omnibus (GEO) database, identified differentially expressed genes (DEGs) by Limma, and selected important module genes *via* weighted gene co-expression network analysis (WGCNA). Functional enrichment analysis, construction of protein–protein interaction network, application of machine learning (least absolute shrinkage and selection operator (LASSO) and random forest (RF)) algorithms, immune cell infiltration analysis, evaluation of nomogram, and receiver operating characteristic (ROC) curve evaluation were subsequently performed to identify pivotal immune-related diagnostic biomarkers for AVC with MS. This research could lead to the identification of immune-associated potential diagnostic markers for AVC in MS patients.

## 2. Materials and Methods

### 2.1 Microarray Data

**Figure 1:**
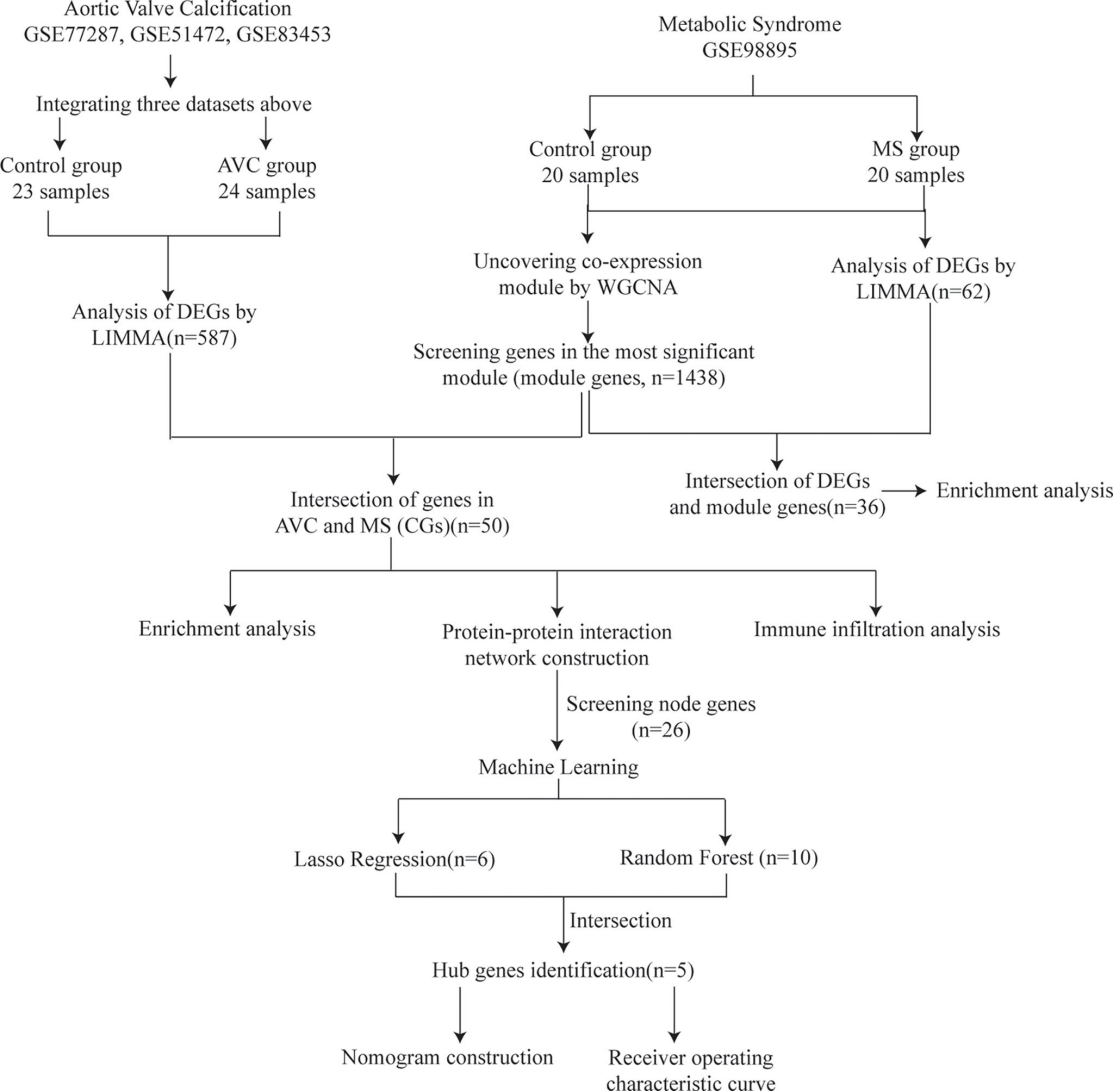
Study flowchart. GSE, gene expression omnibus series; WGCNA, weighted gene co-expression network analysis; Limma, linear models for microarray data; DEGs, differentially expressed genes.

**Figure 1** depicts the study flowchart. Three raw datasets (GSE51472 (13), GSE12644 (14), and GSE83453 (15)) including gene expression data for AVC patients and controls and one dataset of MS (GSE98895 (16)) were downloaded from the GEO (https://www.ncbi.nlm.nih.gov/geo/) database (17). **Table 1** presents detailed dataset information, including the microarray platform, sample groups, and numbers.

**Table 1:** Basic information of GEO datasets used in the study.

GSE series	Type	Sample size		Platform
		Control	Aortic valve calcification	
GSE51472	mRNA	5	5	GPL570
GSE12644	mRNA	10	10	GPL570
GSE83453	mRNA	8	9	GPL10558
		**Control**	**Metabolic syndrome**	
GSE98895	mRNA	20	20	GPL6947

GEO, gene expression omnibus.

### 2.2 Data Processing and Differentially Expressed Gene Screening

First, background calibration, normalization, and log2 transformation were performed on the three AVC raw datasets using affy in R. When multiple probes identified the same gene, the average value was calculated to determine its expression. Following the merging of the three datasets, the Bioconductor “SVA” R package was applied to eliminate batch effects (18). Finally, |log_2_ Fold change (FC)| > 1 (AVC filtration) or 0.585 (MS filtration) and p-value <0.05 were set as the criteria for identifying DEGs using Limma package.

### 2.3 Weighted Gene Co-Expression Network Analysis and Module Gene Selection

WGCNA, a system biology strategy, was adopted to explore the correlation between genes (19). First, the median absolute deviation (MAD) of each gene was determined, and 50% of genes with the smallest MAD were removed. Second, the DEG expression matrix was filtered by the goodSamplesGenes function to omit unqualified genes and samples, and a scale-free co-expression network was built. Third, adjacency was computed using the co-expression similarity-derived “soft” thresholding power (β). The adjacency was then converted into a topological overlap matrix (TOM), and the gene ratio and dissimilarity were determined. The fourth step was the detection of modules using hierarchical clustering and a dynamic tree cut function. Genes with identical expression profiles were classified into gene modules using average linkage hierarchical clustering, with a TOM-based dissimilarity metric and a minimum gene group size (n = 30) for the gene dendrogram. Fifth, the dissimilarity of module eigengenes was computed, a cut line for the module dendrogram was chosen, and several modules were combined for further investigation. The eigengene network was finally visualized. WGCNA analysis was employed to identify important modules in MS.

### 2.4 Functional Enrichment Analysis

The Gene Ontology (GO) system provides structured, computable information regarding the functions of genes and gene products (20). The Kyoto Encyclopedia of Genes and Genomes (KEGG) is a widely used database for the systematic investigation of gene functions (21). Functional enrichment analysis was conducted based on the R package clusterProfiler (22), and the results of enrichment analysis were visualized *via* the Sangerbox platform (http://vip.sangerbox.com/). p-Value <0.05 was set as the criteria. Here, GO and KEGG analyses were performed twice based on the intersection of DEGs and the most significant module genes of MS, and the intersection of DEGs for AVC and the most significant module genes of MS.

### 2.5 Protein–Protein Interaction Network Construction

To excavate interactions among protein-coding genes, a protein–protein interaction (PPI) network was established using the String database (23) (version 11.5; www.string-db.org), with the minimum required interaction score set at 0.400. Cytoscape software was applied to modify images downloaded from String, and an MCODE plug-in was used to identify important interacted genes (24). All genes that could interact with each other in the PPI network were selected for subsequent analysis.

### 2.6 Machine Learning

Two machine learning algorithms were adopted to further filter candidate genes for AVC diagnosis. LASSO is a regression method for selecting a variable to improve the predictive accuracy and is also a regression technique for variable selection and regularization to improve the predictive accuracy and comprehensibility of a statistical model (25). RF is an appropriate approach with the benefits of no limits on variable conditions and better accuracy, sensitivity, and specificity, which can be used to predict continuous variables and provide forecasts without apparent variations (26). “glmnet” (27) and “randomForest” (28) R packages were used to perform LASSO regression and RF analysis. The intersection genes of LASSO and RF were considered as candidate hub genes in AVC diagnosis.

### 2.7 Nomogram Construction and Receiver Operating Characteristic Evaluation

Nomogram construction is valuable for clinical AVC diagnosis. Based on candidate genes, the “rms” R package was applied to construct the nomogram (29). “Points” indicates the score of candidate genes, and “Total Points” indicates the summation of all the scores of genes above. The ROC was subsequently established to evaluate the diagnostic value of candidate genes and nomogram regarding AVC diagnosis, and the calculation of area under the curve (AUC) and 95% CI was performed to quantify its value. AUC > 0.7 was considered the ideal diagnostic value. To further excavate the interrelation among the identified genes, the network was constructed using the String online tool.

### 2.8 Immune Infiltration Analysis

CIBERSORT, a computational approach for identifying the proportion of diverse immune cells using tissue gene expression profiles, was utilized to determine immune cell proportion in AVC and control (30). Immune cell infiltration analysis was performed using the “Cibersort” R package. The barplot was used to visualize the proportion of each type of immune cell in different samples. The comparison regarding the proportion of diverse types of immune cells between AVC and control groups was visualized *via* the vioplot. A heatmap depicting the correlation of 22 types of infiltrating immune cells was carried out using the “corrplot” R package (31).

### 2.9 Statistical Analysis

The establishment of the ROC curve and the calculation of AUC as well as 95% CI were constructed using SPSS Version 26.0 (IBM Corporation, Armonk, NY, USA).

Student’s sample t-test was applied to compare the proportion of different immune cells between the control and AVC groups *via* GraphPad Prism Version 8.3.0 (GraphPad Software, San Diego, CA, USA). p-Value <0.05 was considered statistically significant.

## 3. Results

### 3.1 Identification of Differentially Expressed Genes

**Figure 2:**
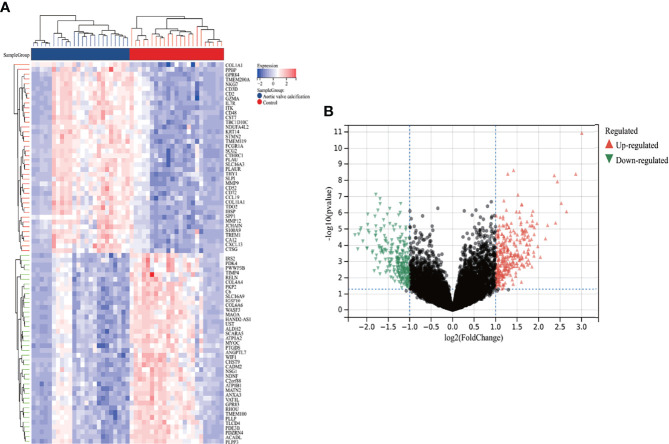
Heatmap and volcano plot for the DEGs identified from the integrated AVC dataset. **(A)** Each row shows the DEGs, and each column refers to one of the samples of AVC cases or controls. The red and blue represent DEGs with upregulated and downregulated gene expression, respectively. **(B)** Red and green plot triangles represent DEGs with upregulated and downregulated gene expression, respectively. AVC, aortic valve calcification; DEGs, differentially expressed genes.

**Figure 3:**
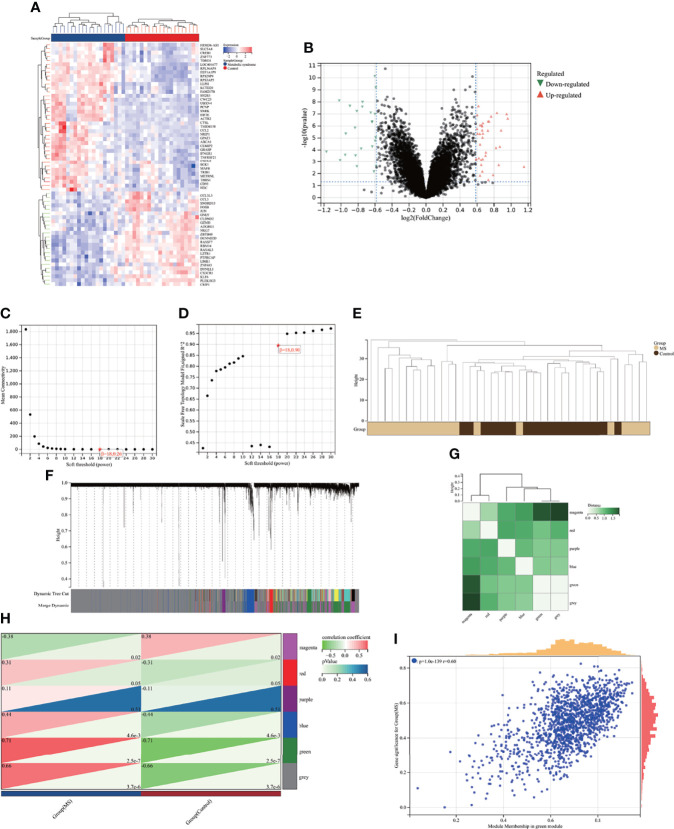
Identification of DEGs *via* Limma and module genes *via* WGCNA in MS. **(A)** The heatmap displays the top 50 upregulated and downregulated DEGs identified from MS dataset. Each row represents the intersection of genes, and each column represents one of MS cases or controls. Red and blue represent upregulated and downregulated gene expression. **(B)** The volcano plot shows all DEGs, of which red and green triangles refer to significant DEGs. **(C, D)** β = 18 is selected as the soft threshold with the combined analysis of scale independence and average connectivity. **(E)** Clustering dendrogram of the MS and control samples. **(F)** Gene co-expression modules represented by different colors under the gene tree. **(G)** Heatmap of eigengene adjacency. **(H)** Heatmap of the association between modules and MS. The green module is shown to be correlated significantly with MS. Numbers at the top and bottom brackets represent the correlation coefficient and p-value, respectively. **(I)** Correlation plot between module membership and gene significance of genes included in the green module. WGCNA, weighted gene co-expression network analysis; Limma, linear models for microarray data; DEGs, differentially expressed genes.

A total of 587 DEGs were identified in the AVC combined dataset using the Limma method, of which 320 were upregulated and 267 were downregulated. The heatmap and volcano plot of AVC DEGs are shown in **Figures 2A, B**. Regarding the MS dataset, 62 DEGs were screened out (38 upregulated and 24 downregulated) (**Figures 3A, B**).

### 3.2 Weighted Gene Co-Expression Network Analysis and Key Module Identification

Here, WGCNA was applied to identify the most correlated module in MS. We chose β = 18 (scale-free R^2^ = 0.9) as the “soft” threshold based on the scale independence and average connectivity (**Figures 3C, D**). **Figure 3E** depicts the clustering dendrogram of the MS and control. On the basis of this power, six gene co-expression modules (GCMs) were generated, which are presented in **Figures 3F, G** in different colors. The correlation between MS and GCMs is shown in **Figure 3H**, and the green module (1,436 genes) demonstrated the highest correlation with MS (correlation coefficient = 0.71, p = 2.5 * 10^−7^) and was regarded as the pivotal module for subsequent analysis. Additionally, we calculated the correlations between module membership and gene significance in the green module for MS. As expected, a significant positive correlation was observed between them (r = 0.6) as shown in **Figure 3I**. Therefore, green module genes were most significantly associated with MS.

### 3.3 Functional Enrichment Analysis of Metabolic Syndrome

**Figure 4:**
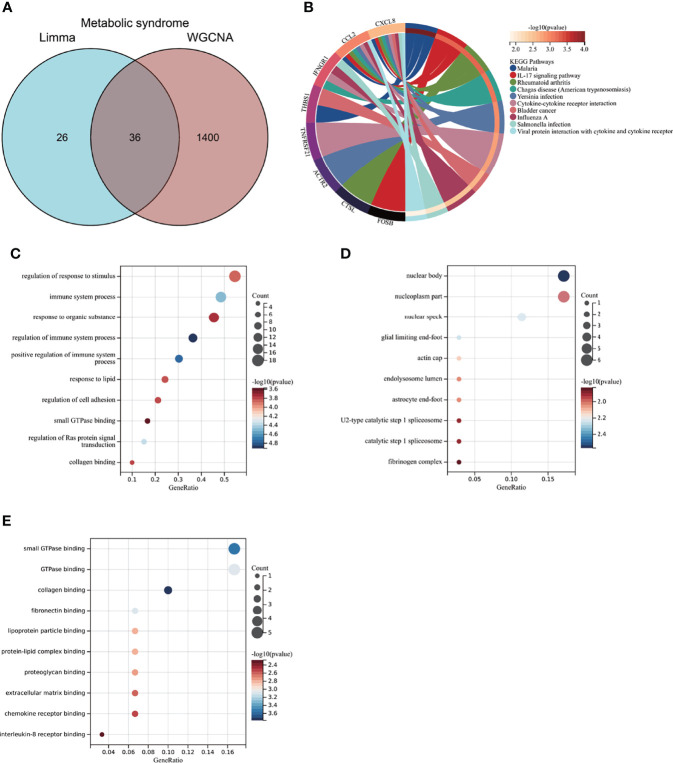
Enrichment analysis of the intersection of genes in MS. **(A)** Venn diagram shows that 36 genes are identified from the intersection of DEGs *via* Limma and green module genes *via* WGCNA. **(B)** KEGG pathway analysis of the intersection of genes. Different colors represent various significant pathways and related enriched genes. **(C–E)** GO analysis of the intersection of genes, including biological process, cellular component, and molecular function, respectively. The y-axis represents different GO terms, the x-axis represents gene ratio enriched in relative GO terms, the circle size refers to gene numbers, and the color represents p-value. MS, metabolic syndrome; KEGG, Kyoto Encyclopedia of Genes and Genomes; GO, Gene Ontology; WGCNA, weighted gene co-expression network analysis; Limma, linear models for microarray data; DEGs, differentially expressed genes.

GSE98895 is a new MS dataset that has not been excavated before. To assess whether this dataset could reflect MS pathogenesis to a reliable extent, we further performed functional enrichment analysis based on the intersection of genes from Limma and WGCNA module genes. A total of 36 common genes (CGs) were screened out *via* the intersection of 62 DEGs and 1436 genes in the green module (**Figure 4A**).

KEGG analysis showed that CGs were primarily enriched in the “IL-17 signaling pathway” and “rheumatoid arthritis” (**Figure 4B**). GO analysis elucidated that CGs were mainly enriched in biological process (BP) terms, including “regulation/positive regulation of immune system process” and “immune system process” (**Figure 4C**). With regard to cellular component (CC) ontology, the CGs were mainly located in the “nuclear body,” “glial limiting end-foot,” and “nuclear speck” (**Figure 4D**). Molecular function (MF) analysis showed that “collagen binding,” “small GTPase binding,” and “fibronectin binding” were the most significant items in CGs (**Figure 4E**). The detailed top ten enrichment ontologies for GO and KEGG are listed in **Supplementary Table S1**.

The enrichment analysis revealed that CGs of MS were mainly related to immune response and inflammatory response, which were highly correlated with MS pathogenesis and reliable for subsequent AVC analysis.

### 3.4 Enrichment Analysis of Aortic Valve Calcification With Metabolic Syndrome and Node Gene Identification *via* Protein–Protein Interaction Network Construction

**Figure 5:**
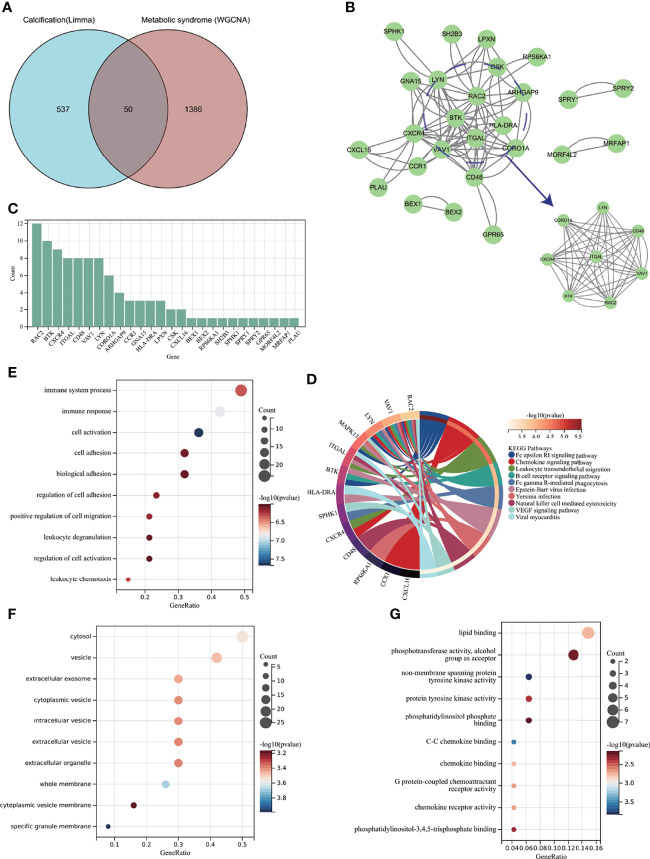
Enrichment analysis of common genes from AVC with MS and the identification of node genes from PPI network. **(A)** Venn diagram shows that 50 common genes are identified from the intersection of genes in AVC using Limma and MS using WGCNA. **(B)** PPI network reveals that 26 genes interact with each other, and the most significant module is visualized using MCODE plug-in. **(C)** The column shows the gene nodes of 26 genes in PPI network. **(D)** KEGG analysis of 50 common genes. **(E–G)** GO analysis (biological process, cellular component, and molecular function) of 50 common genes. AVC, aortic valve calcification; MS, metabolic syndrome; PPI, protein-protein interaction network; WGCNA, weighted gene co-expression network analysis; MCODE, molecular complex detection.

To further explore whether MS-associated pivotal genes could be related to AVC pathogenesis, 50 genes were identified from the intersection of DEGs from AVC and module genes from MS visualized *via* the Venn diagram (**Figure 5A**). The KEGG enrichment analysis revealed that 50 genes were primarily enriched in the “Fc epsilon RI signaling pathway,” “Leukocyte transendothelial migration,” and “Chemokine signaling pathway”; all of the above ontologies were intimately related to the immune system (**Figure 5D**). Also, GO analysis showed that genes were enriched in “cell activation,” “immune response,” and “immune system process” (BP); “specific granule membrane,” “whole membrane,” and “cytosol” (CC); and “non-membrane spanning protein tyrosine kinase activity,” “C-C chemokine binding,” and “lipid binding” (MF) (**Figures 5E–G**, **Supplementary Table S2**).

After confirming that the screened genes were closely related to immunity, we constructed a PPI network to find node genes that could interact with each other for the subsequent machine learning filtration. **Figure 5B** shows the PPI network and that 26 genes could interact with each other; the most active module was visualized through the MCODE plug-in, and the genes were ranked by node numbers in **Figure 5C**.

### 3.5 Identification of Candidate Hub Genes *via* Machine Learning

**Figure 6:**
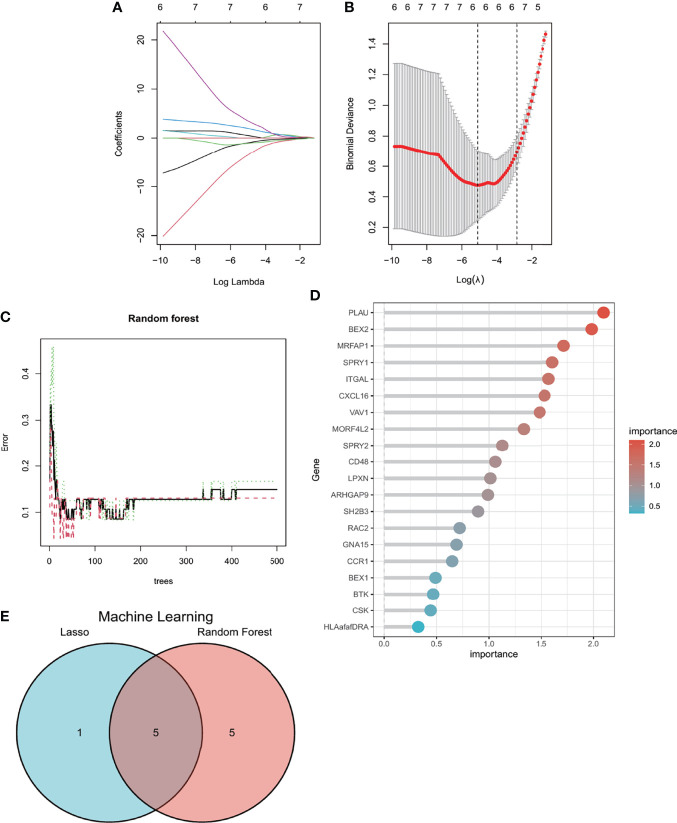
Machine learning in screening candidate diagnostic biomarkers for AVC with MS. **(A, B)** Biomarkers screening in the Lasso model. The number of genes (n = 6) corresponding to the lowest point of the curve is the most suitable for AVC with MS diagnosis. **(C, D)** The random forest algorithm shows the error in AVC; control group and genes are ranked based on the importance score. **(E)** Venn diagram shows that five candidate diagnostic genes are identified *via* the above two algorithms. AVC, aortic valve calcification; MS, metabolic syndrome.

LASSO regression and RF machine learning algorithms were applied to screen candidate genes for nomogram construction and diagnostic value evaluation. As we can see from **Figures 6A, B**, the LASSO regression algorithm identified six potential candidate biomarkers, and the RF algorithm ranked the genes based on the calculation of the importance of each gene (**Figures 6C, D**). The intersection of the top 10 most important genes from the RF and six potential candidate genes from LASSO was visualized *via* the Venn diagram (**Figure 6E**), and five genes (*BEX2*, *CXCL16*, *ITGAL*, *MORF4L2*, and *SPRY2*) were identified for the final validation. Moreover, based on the five genes, we found that *CXCL16*, *ITGAL*, *MORF4L2*, and *SPRY2* could interact with each other through intermediate molecules, while *BEX2* showed a different pattern (**Supplementary Figure S1**).

### 3.6 Diagnostic Value Assessment

**Figure 7:**
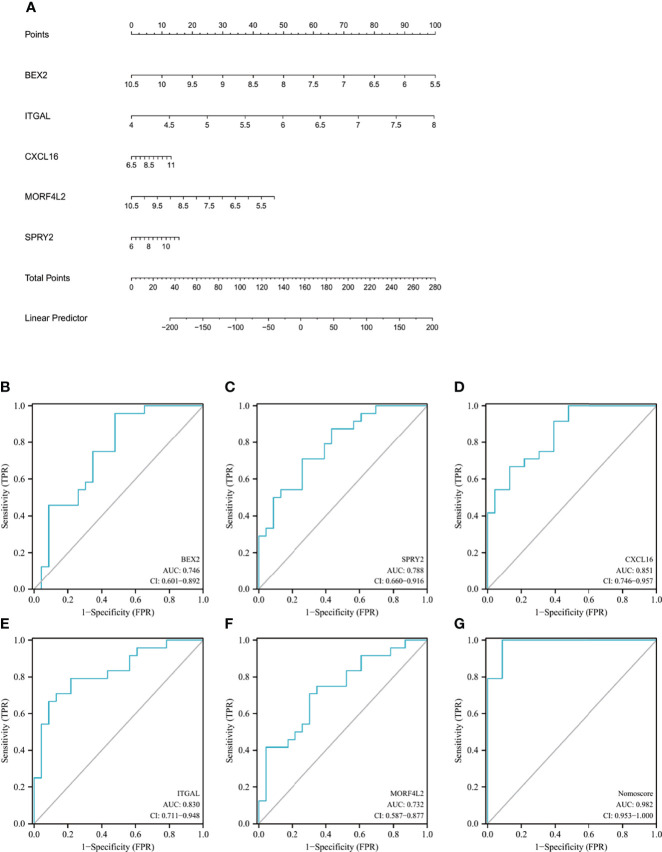
Nomogram construction and the diagnostic value evaluation. **(A)** The visible nomogram for diagnosing AVC with MS. **(B–G)** The ROC curve of each candidate gene (*BEX2*, *SPRY2*, *CXCL16*, *ITGAL*, and *MORF4L2*) and nomogram show the significant AVC with MS diagnostic value. AVC, aortic valve calcification; MS, metabolic syndrome; BEX2, brain expressed X-linked 2; SPRY2, sprouty RTK signaling antagonist 2; CXCL16, C-X-C motif chemokine ligand 16; ITGAL, integrin subunit alpha L; MORF4L2, mortality factor 4 like 2; AUC, area under the curve.

The nomogram was constructed based on the five candidate hub genes (**Figure 7A**), and a ROC curve was established to assess the diagnostic specificity and sensitivity of each gene and the nomogram. We calculated the AUC and 95% CI for each item. The results were as follows: *BEX2* (AUC 0.746, CI 0.601–0.892), *SPRY2* (AUC 0.788, CI 0.660–0.916), *CXCL16* (AUC 0.851, CI 0.746–0.957), *ITGAL* (AUC 0.830, CI 0.711–0.948), *MORF4L2* (AUC 0.732, CI 0.587–0.877), and nomogram (AUC 0.982, CI 0.953–1.000) (**Figures 7B–G**). All the candidate genes possess a high diagnostic value for AVC with MS, and the constructed nomogram had the highest diagnostic value.

### 3.7 Immune Cell Infiltration Analysis

Since we observed that MS-associated genes could regulate AVC pathogenesis and were mainly enriched in immune regulation and could be used as the potential AVC diagnostic biomarker by nomogram construction with ROC evaluation, immune cell infiltration analysis was performed to better elucidate the immune regulation of AVC.

**Figure 8:**
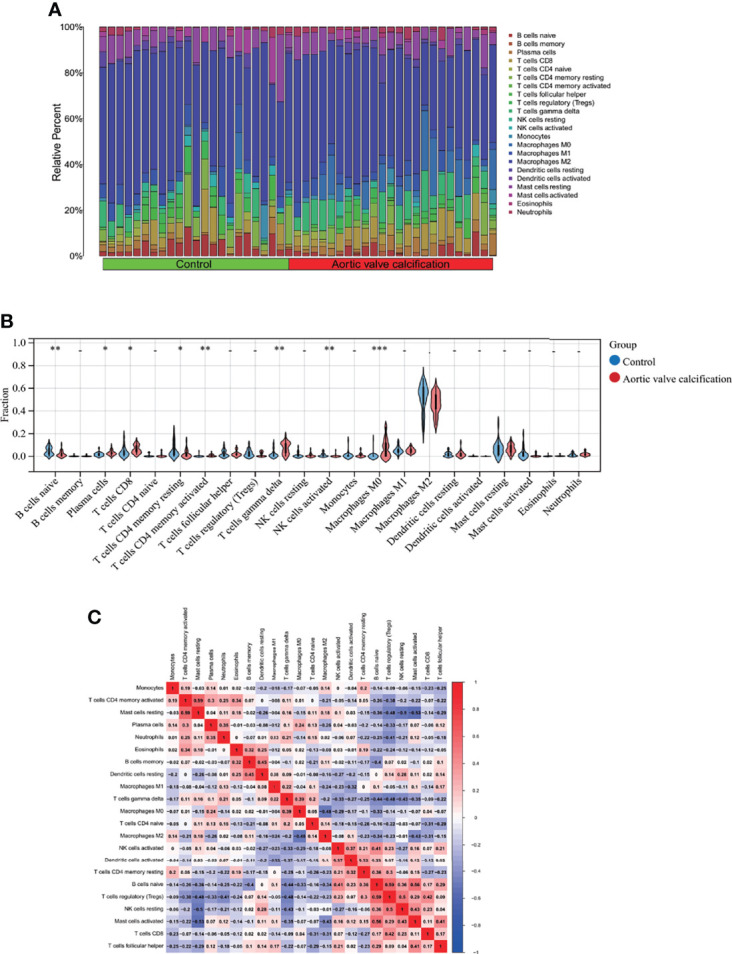
Immune cell infiltration analysis between AVC and control. **(A)** The proportion of 22 kinds of immune cells in different samples visualized from the barplot. **(B)** Comparison regarding the proportion of 22 kinds of immune cells between AVC and control groups visualized by the vioplot. **(C)** Correlation of 22 immune cell type compositions. *, p < 0.05, **, p < 0.01, ***, p < 0.001. Both horizontal and vertical axes demonstrate immune cell subtypes. AVC, aortic valve calcification.

Regarding the AVC and control groups, the proportion of 22 kinds of immune cells in each sample is displayed in the barplot (**Figure 8A**). The vioplot demonstrated that AVC patients had a higher level of CD8^+^ T cells, plasma cells, CD4 memory activated T cells, and M0 macrophages and a lower level of naive B cells, CD4 resting T cells, activated NK cells, and M2 macrophages (**Figure 8B**). The correlation of 22 types of immune cells revealed that CD4 memory activated T cells were positively associated with resting mast cells (r = 0.59) and that naive B cells were positively related to activated mast cells (r = 0.56), whereas resting mast cells were negatively related to resting NK cells (r = −0.50) (**Figure 8C**). In general, various kinds of immune cells were differentially infiltrated in AVC patients, which could serve as the potential regulation point for AVC treatment.

## 4. Discussion

AVC is a major cause of cardiac dysfunction in the elderly population and leads to a great public health burden. Recent studies have identified several new biomarkers for AVC diagnosis, such as matrix remodeling associated with protein 5, fibronectin type III domain containing 1, and miR-34a (32, 33). There have been no previous studies that have combined the two diseases. Furthermore, machine learning methods and nomogram generation have not been used in the diagnosis of AVC. Here, we used a series of integrated bioinformatics analysis and machine learning methods to construct the nomogram and to evaluate the diagnostic value for AVC in MS patients. The most noteworthy discovery is that we identified five pivotal immune-associated candidate genes (*ITGAL*, *CXCL16*, *BEX2*, *SPRY2*, and *MORF4L2*) and developed a nomogram for diagnosing AVC in MS patients.

Samples regarding the MS dataset used in the study are all from peripheral blood; therefore, we only need to collect peripheral blood from MS patients and evaluate the expression of the five discovered immune-associated genes to infer the probability of AVC incidence in MS patients, which is an efficient and practical method for clinical usage. The application of peripheral blood tests in diagnosing different diseases has also been widely accepted (34, 35). Moreover, although we confirmed that gene expression level can be used as an independent diagnostic marker, we intend to develop a more comprehensive diagnosis pattern by converting it into a score and taking all these five markers into consideration (36). The expression of each gene was quantified and transferred to a score, with the augmentation of the score referring to the linear predictor. We can monitor and intervene early in the MS patients when the linear predictor is high, which is more valuable for implementation in AVC with MS diagnosis.

Integrin alpha L (*ITGAL*) is the novel biomarker identified in our study in diagnosing AVC in MS patients. It belongs to the integrin family and is also known as CD11a. It serves as the receptor for the intercellular adhesion molecule (ICAM) family (*ICAM1*, *ICAM2*, *ICAM3*, and *ICAM4*) *(* 37) as well as the secreted form of ubiquitin-like protein *ISG15 (* 38). It is predominantly expressed in immune cells and is involved in numerous immunological phenomena, such as leukocyte–endothelial cell contact, cytotoxic T cell-mediated killing, and antibody-dependent killing by granulocytes and monocytes (39). It promotes apoptotic neutrophil phagocytosis by macrophages together with *ICAM3 (* 40). It is involved in immunological reactions and inflammatory processes, as well as angiogenesis and cancer growth. It has been identified as a biomarker in diverse cancers, including gastric, ovarian, colorectal, and renal cancers (41–43). However, the mechanisms of *ITGAL* regarding immune engagement with AVC remain unclear. Some AVC risk factors enhance the production of adhesion molecules such as ICAM in valvular endothelial cells. Pulsatile shear stress caused elevated *ICAM-1* levels in aortic explanted leaflets (44). Wang et al. (45) discovered that ICAM signaling is involved in decreased osteogenic bone morphogenic protein and ALP alkaline phosphatase levels in valvular interstitial cells, which are engaged in calcific valve rebuilding. As a receptor of multiple ICAM family members, *ITGAL* is also overexpressed in AVC. Thus, we suggest that *ITGAL* could represent a potential diagnostic target for AVC in MS patients.

C-X-C motif chemokine ligand 16 (*CXCL16*) may serve a pro-inflammatory function in human atherosclerosis, particularly in acute coronary syndrome (46). Its expression was shown to be considerably elevated in atherosclerotic plaque, and it participates in mechanisms that lead to increased stenosis in atherosclerotic coronary arteries (47). During inflammatory valvular heart disease, *SR-PSOX/CXCL16* is engaged in the recruitment of CD8^+^ T cells *via* activating *VLA-4* and stimulating IFN-γ production (48). Studies have demonstrated that cytokine signaling and small dense low-density lipoprotein (LDL) particles play a role in the fibrotic and calcific remodeling of AVC. Our study found that *CXCL16* was overexpressed in the AVC with MS patients; thus, we surmise that *CXCL16* may induce CD8^+^ T-cell infiltration and ox-LDL metabolism, resulting in the AVC process. It can also be used as a diagnostic biomarker.

Brain expressed X-linked 2 (*BEX2*) has been shown to control mitochondrial apoptosis and the G1 cell cycle in breast cancer (49) and to increase the proliferation of human glioblastoma cells (50). Sprouty RTK Signaling Antagonist 2 (*SPRY2*) is involved in cell proliferation and differentiation and can modulate receptor tyrosine kinase signaling. By inhibiting FGFR2-induced ERK phosphorylation, Xu et al. (51) found that *SPRY2* was associated with a favorable prognosis for intrahepatic cholangiocarcinoma. Mortality Factor 4 Like 2 (*MORF4L2*) is a component of the NuA4 histone acetyltransferase complex, which is involved in transcriptional activation of select genes primarily by acetylating nucleosomal histone H4 and H2A (52).

Subsequently, we explored the interrelation among the five identified immune-associated genes. As depicted in **Supplementary Figure S1**, Intercellular Adhesion Molecule 1 (*ICAM1*) acts as a bridge by binding *CXCL16* and *ITGAL* directly. Additionally, previous research has clarified the role of the *CXCL16/ICAM1/ITGAL* pathway in the regulation of inflammation and immunological diseases. First, *ICAM1* is the ligand for *ITGAL*, and stabilizing the *ITGAL/ICAM1* complex is crucial for the regulation of the adaptive immunological process (53). Second, it has been confirmed that the regulation of *CXCL16* is closely related to *ICAM1* in numerous diseases. Abu et al. (54) reported that treatment of human retinal microvascular endothelial cells (HRMECs) with *CXCL16* led to increased production of *ICAM-1* and increased leukocyte adherence to HRMECs, which led to an inflammatory response. Zhao et al. (55) discovered that *CXCL16* could impact the establishment of atherosclerotic lesions by targeting *ICAM1*. Taking into account the network and prior findings, *CXCL16* may regulate many immunological and inflammatory processes *via* indirect interactions with *ITGAL*. The relationship between *MORF4L2* and *SPRY2* is not as close as the relationship between *CXCL16* and *ITGAL*. Based on the shortest link path, the bridge is composed of Lysine Acetyltransferase 5 (*KAT5*), Tumor Protein P53 (*TP53*), and Erb-B2 Receptor Tyrosine Kinase 3 (*ERBB3*). *KAT5* is a histone acetylase that is crucial for regulating apoptosis, autophagy, RNA transcription, and circadian rhythms (56) and for activating *TP53* in relation to tumor DNA repair (57). *ERBB3* is a receptor tyrosine kinase of the epidermal growth factor receptor (EGFR) family. Zhang et al. (58) discovered that activating *ERBB3* could mitigate myocardial ischemia/reperfusion damage. Due to the lengthy pathway, the association between *MORF4L2* and *SPRY2* is inconclusive; further research is required to determine whether the two genes regulate the immunological process in a comparable pattern.

Previous studies have demonstrated that immunological modulation and inflammatory modulation appear in all stages of AVC. Abdelbaky et al. (59) discovered that early AV inflammation may predispose patients to AV sclerosis based on 111 participants. Coté et al. (60) performed a histological analysis in 285 patients with AVC undergoing AV replacement and analyzed the presence of chronic inflammatory infiltrates. They found that dense inflammatory infiltrates within AVC are associated with the severity of aortic stenosis. Additionally, Mazzone et al. (61) found that neo-angiogenesis, T-lymphocyte infiltration, and heat shock protein-60 are biological hallmarks of an immune-mediated inflammatory process in end-stage AVC. According to our results, AVC patients had a higher level of CD8^+^ T cells, plasma cells, CD4 memory activated T cells, and M0 macrophages and a lower level of naive B cells, CD4 resting T cells, activated NK cells, and M2 macrophages, which are consistent with the previous studies. Above all, understanding inflammatory signaling mechanisms can pave the way for the development of diagnosis and targeted therapeutics of AVC.

## 5. Limitation

Our study had several limitations. First, although we pooled three AVC datasets, the samples still remained few, and the diagnostic value of the nomogram was rather high due to the limited sample size. Also, we aimed to choose another dataset for validating the diagnostic value. However, only one dataset with only six samples was available. Thus we were unable to validate the findings. The results should be subsequently confirmed in a more large-scale study with a large sample size. Second, although the five candidate hub genes were mainly enriched in regulating immune pathways, the interaction between candidate hub genes and dysregulated immune cells was still worth investigating.

## 6. Conclusion

Our study systematically discovered five immune-associated candidate hub genes (*ITGAL*, *CXCL16*, *MORF4L2*, *SPRY2*, and *BEX2*) and provided the nomogram for diagnosing AVC with MS by various bioinformatics analysis and machine learning algorithms. We also point out the dysregulated immune cell proportion in AVC with MS. Our study could provide potential peripheral blood diagnostic candidate genes for AVC in MS patients.

## Data Availability Statement

The datasets presented in this study can be found in online repositories. The names of the repository/repositories and accession number(s) can be found in the article/**Supplementary Material**.

## Author Contributions

JW and KW contributed to the hypothesis development and manuscript preparation. YZ, WS, and DZ contributed to the study design and data analysis. SX contributed to the image processing. All the authors drafted the manuscript and approved its submission.

## Conflict of Interest

The authors declare that the research was conducted in the absence of any commercial or financial relationships that could be construed as a potential conflict of interest.

## Publisher’s Note

All claims expressed in this article are solely those of the authors and do not necessarily represent those of their affiliated organizations, or those of the publisher, the editors and the reviewers. Any product that may be evaluated in this article, or claim that may be made by its manufacturer, is not guaranteed or endorsed by the publisher.

## Supplementary Material

Supplementary Table 1 Functional enrichment analysis of intersection genes *via* Limma and WGCNA in metabolic syndrome.

Supplementary Table 2 Functional enrichment analysis of differentially expressed genes in aortic valve calcification with metabolic syndrome.

Supplementary Figure 1 The interaction among five identified immune associated genes in diagnosing aortic valve calcification with metabolic syndrome.
