# Supplementary material for: Identification of Immune-Associated Genes in Diagnosing Aortic Valve Calcification With Metabolic Syndrome by Integrated Bioinformatics Analysis and Machine Learning
Source: Front Immunol. 2022 Jul 4;13:937886. doi: 10.3389/fimmu.2022.937886 (PMC9295723; doi:10.3389/fimmu.2022.937886)

**Supplementary Table S1.** Functional enrichment analysis of intersection genes via Limma and WGCNA in metabolic syndrome.

| Term | Count | adj. P value | Genes |
| --- | --- | --- | --- |
| **Biological Process** |  |  |  |
| regulation of immune system process | 12 | 0.014 | CXCL8/RASAL3/TRIB1/CCL2/LIME1/RBM14/THBS1/TNFRSF21/  CTSL/MAFB/IFNGR1/ACTR2 |
| positive regulation of immune system process | 10 | 0.014 | CXCL8/RASAL3/TRIB1/CCL2/LIME1/RBM14/THBS1/TNFRSF21/  CTSL/ACTR2 |
| immune system process | 16 | 0.017 | CXCL8/KLF6/RASAL3/TRIB1/CCL2/LIME1/RBM14/THBS1/  TNFRSF21/CTSL/SNRK/SH2B3/MAFB/CRIP1/IFNGR1/ACTR2 |
| regulation of Ras protein signal transduction | 5 | 0.018 | LZTR1/ABCA1/PLEKHG3/RASAL3/ADGRG1 |
| regulation of response to stimulus | 7 | 0.035 | LZTR1/ABCA1/CXCL8/PLEKHG3/RASAL3/TRIB1/CCL2/  ADGRG1/LIME1/RBM14/METRNL/THBS1/TNFRSF21/CTSL/  SH2B3/IFNGR1/GPAT3/ACTR2 |
| response to lipid | 15 | 0.035 | ABCA1/NRIP1/CXCL8/TRIB1/CCL2/RBM14/THBS1/FOSB |
| regulation of cell adhesion | 4 | 0.035 | CXCL8/RASAL3/CCL2/ADGRG1/THBS1/TNFRSF21/SH2B3 |
| response to organic substance | 5 | 0.035 | ABCA1/UBXN4/NRIP1/CXCL8/TRIB1/CCL2/RBM14/THBS1/  TNFRSF21/CTSL/SH2B3/CRIP1/IFNGR1/FOSB/ACTR2 |
| response to unfolded protein | 4 | 0.035 | UBXN4/CXCL8/CCL2/THBS1 |
| regulation of small GTPase mediated signal transduction | 5 | 0.035 | LZTR1/ABCA1/PLEKHG3/RASAL3/ADGRG1 |
| **Cellular Component** |  |  |  |
| nuclear body | 6 | 0.003 | NRIP1/SGK1/CWC25/RBM14/PCNP/EIF3E |
| glial limiting end-foot | 1 | 0.006 | ADGRG1 |
| nuclear speck | 4 | 0.006 | NRIP1/SGK1/CWC25/RBM14 |
| actin cap | 1 | 0.007 | ACTR2 |
| endolysosome lumen | 1 | 0.009 | CTSL |
| astrocyte end-foot | 1 | 0.009 | ADGRG1 |
| nucleoplasm part | 6 | 0.012 | NRIP1/SGK1/CWC25/RBM14/PCNP/EIF3E |
| U2-type catalytic step 1 spliceosome | 1 | 0.013 | CWC25 |
| catalytic step 1 spliceosome | 1 | 0.013 | CWC25 |
| fibrinogen complex | 1 | 0.015 | THBS1 |
| **Molecular Function** |  |  |  |
| collagen binding | 3 | 0.028 | ADGRG1/THBS1/CTSL |
| small GTPase binding | 5 | 0.028 | LZTR1/ABCA1/PLEKHG3/GRASP/DENND2D |
| fibronectin binding | 2 | 0.043 | THBS1/CTSL |
| GTPase binding | 5 | 0.043 | LZTR1/ABCA1/PLEKHG3/GRASP/DENND2D |
| lipoprotein particle binding | 2 | 0.055 | ABCA1/THBS1 |
| protein-lipid complex binding | 2 | 0.055 | ABCA1/THBS1 |
| proteoglycan binding | 2 | 0.055 | THBS1/CTSL |
| extracellular matrix binding | 2 | 0.068 | ADGRG1/THBS1 |
| chemokine receptor binding | 2 | 0.072 | CXCL8/CCL2 |
| interleukin-8 receptor binding | 1 | 0.087 | CXCL8 |
| **KEGG Pathway** |  |  |  |
| Malaria | 3 | 0.007 | CXCL8/CCL2/THBS1 |
| IL-17 signaling pathway | 3 | 0.016 | CXCL8/CCL2/FOSB |
| Rheumatoid arthritis | 3 | 0.016 | CXCL8/CCL2/CTSL |
| Chagas disease (American trypanosomiasis) | 3 | 0.016 | CXCL8/CCL2/IFNGR1 |
| Yersinia infection | 3 | 0.021 | CXCL8/CCL2/ACTR2 |
| Cytokine-cytokine receptor interaction | 4 | 0.024 | CXCL8/CCL2/TNFRSF21/IFNGR1 |
| Bladder cancer | 2 | 0.029 | CXCL8/THBS1 |
| Influenza A | 3 | 0.035 | CXCL8/CCL2/IFNGR1 |
| Salmonella infection | 2 | 0.083 | CXCL8/IFNGR1 |

**Supplementary Table S2.** Functional enrichment analysis of differentially expressed genes in aortic valve calcification with metabolic syndrome.

| Term | Count | adj. P value | Genes |
| --- | --- | --- | --- |
| **Biological Process** |  |  |  |
| cell activation | 17 | 0.000 | PLAU/RAC2/ITGAL/ARHGAP9/CORO1A/CLEC5A/VAV1/  JAML/SLC2A3/LYN/BTK/GNA15/RPS6KA1/SPHK1/CSK/PNP/  SH2B3 |
| immune response | 20 | 0.000 | PLAU/CD48/RAC2/ITGAL/ARHGAP9/CORO1A/CXCL16/  CLEC5A/VAV1/JAML/GPR65/SLC2A3/HLA-DRA/LYN/BTK/  CCR1/CSK/CYBC1/LPXN/PNP |
| immune system process | 23 | 0.000 | PLAU/CD48/RAC2/ITGAL/ARHGAP9/CORO1A/CXCL16/  CLEC5A/VAV1/JAML/GPR65/SLC2A3/HLA-DRA/LYN/BTK/  CCR1/SPHK1/CSK/CYBC1/LPXN/PNP/SH2B3/CXCR4 |
| leukocyte chemotaxis | 7 | 0.000 | RAC2/CORO1A/VAV1/JAML/LYN/CCR1/CXCR4 |
| positive regulation of cell migration | 10 | 0.000 | PLAU/RAC2/CORO1A/CXCL16/SPRY2/LYN/CCR1/SPHK1/  CXCR4/SPAG9 |
| regulation of cell adhesion | 11 | 0.000 | PLAU/RAC2/CORO1A/PDE3B/VAV1/LYN/CSK/LPXN/PNP/  SH2B3/CXCR4 |
| cell adhesion | 15 | 0.000 | PLAU/RAC2/ITGAL/CORO1A/PDE3B/VAV1/JAML/LYN/CCR1/  TNFRSF12A/CSK/LPXN/PNP/SH2B3/CXCR4 |
| leukocyte degranulation | 10 | 0.000 | PLAU/RAC2/ITGAL/ARHGAP9/CORO1A/CLEC5A/SLC2A3/  LYN/BTK/PNP |
| biological adhesion | 15 | 0.000 | PLAU/RAC2/ITGAL/CORO1A/PDE3B/VAV1/JAML/LYN/CCR1/  TNFRSF12A/CSK/LPXN/PNP/SH2B3/CXCR4 |
| regulation of cell activation | 10 | 0.000 | RAC2/CORO1A/VAV1/LYN/BTK/RPS6KA1/SPHK1/CSK/PNP/  SH2B3 |
| **Cellular Component** |  |  |  |
| specific granule membrane | 4 | 0.012 | PLAU/ITGAL/CLEC5A/SLC2A3 |
| whole membrane | 13 | 0.012 | PLAU/CD48/RAC2/ITGAL/CORO1A/CLEC5A/SLC2A3/HLA-DRA/  LYN/BTK/SPHK1/CSK/SYT11 |
| cytosol | 25 | 0.012 | RAC2/ARHGAP9/CORO1A/PDE3B/CLEC5A/MAPK13/VAV1/  BCAT1/FBP1/SPRY2/LYN/BTK/RPS6KA1/SPHK1/OSBPL3/  SPRY1/CSK/LPXN/FAM13B/AHCTF1/PNP/SH2B3/POLR2H/  SPAG9/MRFAP1 |
| vesicle | 21 | 0.012 | PLAU/CD48/RAC2/ITGAL/ARHGAP9/CORO1A/CLEC5A/FBP1/  SLC2A3/HLA-DRA/LYN/BTK/AOAH/SPHK1/CSK/METRNL/  AHCTF1/PNP/CXCR4/SYT11/SPAG9 |
| extracellular exosome | 15 | 0.012 | PLAU/CD48/RAC2/ITGAL/CORO1A/FBP1/SLC2A3/HLA-DRA/  LYN/CSK/METRNL/AHCTF1/PNP/CXCR4/SPAG9 |
| cytoplasmic vesicle | 15 | 0.012 | PLAU/RAC2/ITGAL/ARHGAP9/CORO1A/CLEC5A/SLC2A3/  HLA-DRA/BTK/AOAH/SPHK1/PNP/CXCR4/SYT11/SPAG9 |
| intracellular vesicle | 15 | 0.012 | PLAU/RAC2/ITGAL/ARHGAP9/CORO1A/CLEC5A/SLC2A3/  HLA-DRA/BTK/AOAH/SPHK1/PNP/CXCR4/SYT11/SPAG9 |
| extracellular vesicle | 15 | 0.012 | PLAU/CD48/RAC2/ITGAL/CORO1A/FBP1/SLC2A3/HLA-DRA/  LYN/CSK/METRNL/AHCTF1/PNP/CXCR4/SPAG9 |
| extracellular organelle | 15 | 0.012 | PLAU/CD48/RAC2/ITGAL/CORO1A/FBP1/SLC2A3/HLA-DRA/  LYN/CSK/METRNL/AHCTF1/PNP/CXCR4/SPAG9 |
| cytoplasmic vesicle membrane | 8 | 0.018 | PLAU/RAC2/ITGAL/CORO1A/CLEC5A/SLC2A3/HLA-DRA/SYT11 |
| **Molecular Function** |  |  |  |
| non-membrane spanning protein tyrosine kinase activity | 3 | 0.033 | LYN/BTK/CSK |
| C-C chemokine binding | 2 | 0.033 | CCR1/CXCR4 |
| lipid binding | 7 | 0.089 | ARHGAP9/SESTD1/LYN/BTK/SPHK1/OSBPL3/PGRMC2 |
| chemokine binding | 2 | 0.089 | CCR1/CXCR4 |
| G protein-coupled chemoattractant receptor activity | 2 | 0.089 | CCR1/CXCR4 |
| chemokine receptor activity | 2 | 0.089 | CCR1/CXCR4 |
| **KEGG Pathway** |  |  |  |
| Fc epsilon RI signaling pathway | 5 | 0.000 | RAC2/MAPK13/VAV1/LYN/BTK |
| Chemokine signaling pathway | 6 | 0.001 | RAC2/CXCL16/VAV1/LYN/CCR1/CXCR4 |
| Leukocyte transendothelial migration | 5 | 0.001 | RAC2/ITGAL/MAPK13/VAV1/CXCR4 |
| B cell receptor signaling pathway | 4 | 0.004 | RAC2/VAV1/LYN/BTK |
| Fc gamma R-mediated phagocytosis | 4 | 0.006 | RAC2/VAV1/LYN/SPHK1 |
| Epstein-Barr virus infection | 5 | 0.009 | ITGAL/MAPK13/HLA-DRA/LYN/BTK |
| Yersinia infection | 4 | 0.011 | RAC2/MAPK13/VAV1/RPS6KA1 |
| Natural killer cell mediated cytotoxicity | 4 | 0.012 | CD48/RAC2/ITGAL/VAV1 |
| VEGF signaling pathway | 3 | 0.012 | RAC2/MAPK13/SPHK1 |
| Viral myocarditis | 3 | 0.012 | RAC2/ITGAL/HLA-DRA |

**Supplementary Figure S1.** The interaction among five identified immune-associated genes in diagnosing aortic valve calcification with metabolic syndrome.


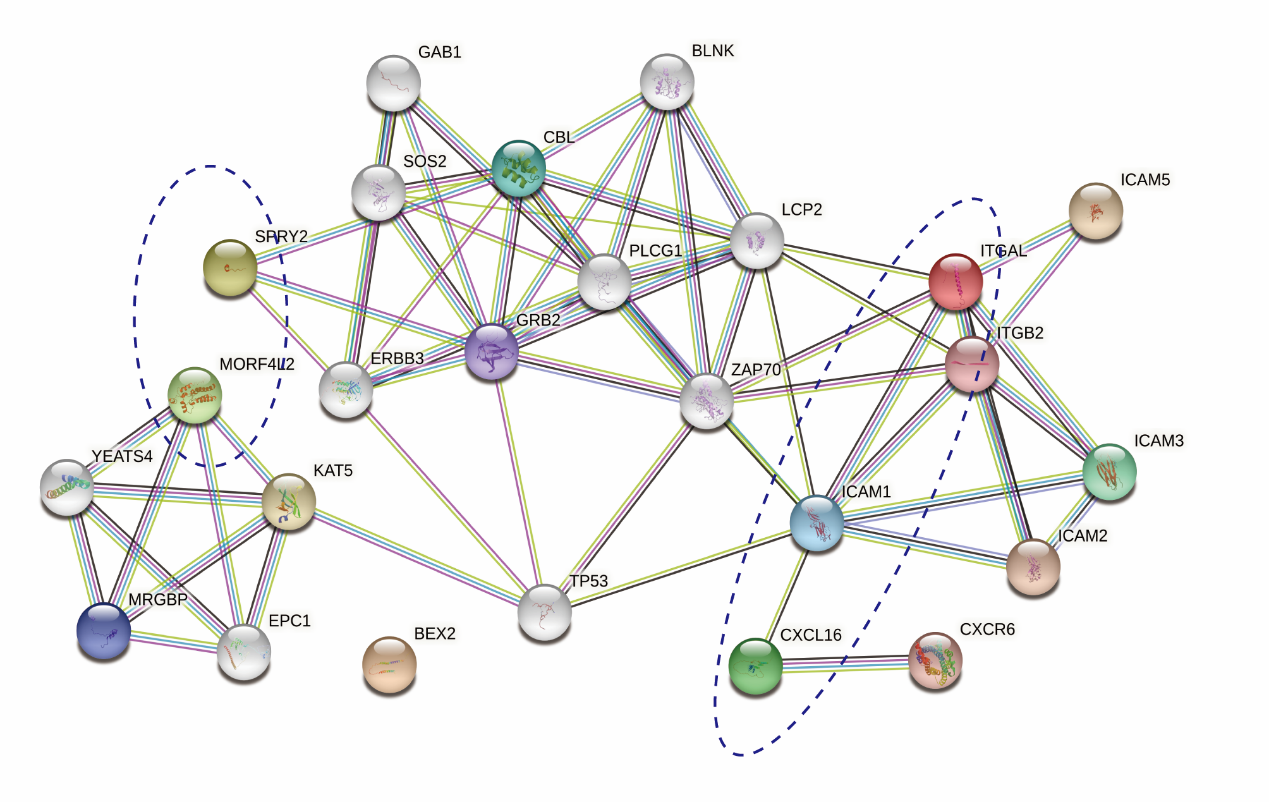

Supplement: Supplementary Table 1 — Functional enrichment analysis of intersection genes via Limma and WGCNA in metabolic syndrome. [file DataSheet_1.docx]
